# Locating the Association

**Published:** 1885-10

**Authors:** R. J. Nunn

**Affiliations:** Savannah, Ga.


					﻿Correspondence.
LOCATING THE ASSOCIATION.
Editors Atlanta Medical and Surgical ‘Journal:
With the exception of the items relating to the membership of
the Association which are taken from the proceedings of 1884
the information embodied in the following tables is derived
from the “ Georgia State Gazetteer,” vol. iii, 1883-4, an<^ cannot
fail to be of great value, not only to the committee, but also to
physicians seeking for locations, of which an inspection of the
tables will show there is no lack in Georgia. The Gazetteer gives
a directory of certain towns or districts. Wherever this is done
the name of that town or district has been incorporated in the
following tables :
Table No. I.
This table gives the names of 1,201 towns or districts, the
county in which each town is located, the number of inhabitants,
the number of physicians and the number of members of the
Association.
CT* cj	<» C3
, .2 _q o	u.2 .a o
c	a
towv COUNTY, g ago'S TOWN. COUNTY. §	° S
1OWN.	.2	.2
—	£1 -C	'■* Xi ”2
o. 5	g. a
00	00
P-i H	<3	Ch H	<5
Abbeville.....Wilcox....	150	1.... Albany........Dough’ty.	4000	5	3
Absalom....... Hall............. .... Alexander.....Burke......	200	1 ..
Acree......... Worth................. Algernon......Gwinnett.	100......
Acworth.......Cobb.......	700	3	1 Alice........ Pulaski..............
Adairsville...Bartow....	500	3.... Allandale..... Banks.....	150......
Adamsville....Fulton ....	150	2 ... Allapaha ..... Berrien ...	500	1 ..
Adel..........Berrien...	150	1.... Allatoona.....Bartow....	100.........
Aerial........Hab’sham 1200.......... Allens........ Ricbm’d.......... 1..
Afton.........Berrien....	50........ Allen’s Mills.Carroll....	500	.....
Agate.........Floyd......	250	...... Alkgator .....Telfair....	300	.....
Air Line......Hart.......	200	1 ... Alpharetta....Milton.....	250	2..
Alaculsy......Murray....	300........ Alpine........Chattooga	300	3..
Table No. I.—Continued.
a .s	c3
| o	2;s
^>. c	®	C
TOWN. COUNTY, g a£ ©S TOWN. COUNTY. g g£
.2	©	®<i	.2	5*	“<!
3	*	3	«	5^
g.	5	g.	3
o	o	O	o	**
H	3	•	fe	H	S
Altamaha........Tattnall................. Bartow...........Jefferson.. 500	5	1
Alto............Banks.....	100......... Bartow I’n Wks Bartow......	200 ........
Amandaville.....Hart......	150......... Bascom...........Scriven.... 200 ...... .
Americus........Sumter ....	5000	8	4 Bath............ Richm’d ..	125	1.....
Amicolola.......Dawson ...	150........ Battle Ground .. Johnson...	300	1 ....
Andersonville... Sumter....	300	2	1 Baxley.......... Appling.. 250	4 ....
Antioch.........Troup.....	150	2 ..... Bay Branch....... Emanuel.	75........
Anvil Block.....Henry.....	100........ Bay Creek........Gwinnett.	75........
Apex............Mitchell................. Beard’s Creek... Liberty.... 150.........
Apple Valley....	Jackson....	125	1..... Beaverdale....... Whitfield.	350	1 ....
Appling.........Columbia. 125	2 ... Belair........... Richm’d ..	80........
Arabi...........Dooly.....	150........ Belleview........ Talbot...	100	1.....
Arcadia.........Liberty.... 200 ......... Belton........... Hall.....	500	2 ....
Arcola..........Bulloch....	100........ Ben. Hill........ Fulton........ 3.......
Argyle..........Clinch....	100........ Benevolence....Randolph	150	1.....
Arlington.......Calhoun..	550	5 .... Berkshire........Gwinnett.......	1.....
Arp.............Banks.....	200	....... Berzelia.........Columbia.	75........
Arthur..........Laurens... 250 .......... Bickley.......... Ware.....	150	2 ....
Asbury..........Troup.....	40	2 ... Big Sandy........Twiggs. ...	200	2 ....
Ascalon.........Walker................... Birdford  Tattnall.... 200 ..............
Astor...........Clayton....	50. Black Creek.............Scriven....	150	1.....
Athens..........Clarke......	8000	9	7	Blackacre... Bulloch....	150	1.....
Athal...........Monroe ...	100.. Blackshear.............Pierce.... 1000	3 ....
Atkinson........Wayne .... 200 .......... Blaine...........Hab’sbam 100............
Atlanta.........Fulton.... 50000	133	43	Blairsville. Union....	200	2 ....
Attapulgus......Decatur..........	1 ... Blakely..........Early..... 1000	4	1
Auburn .........Gwinnett. 300	3 ... Blitch........... Bulloch....... 2.......
Aucilla.........Thomas.... 500	1 ... Bloodsworth’s... Wilk’nson 200 ..........
Augusta.........Richm’d.. 34000	46	20 Blountsville...Jones.....	200	3 ....
Auraria.........Lumpkin. 75.............. Bloys............ Bulloch.... 200 .......
Ava.............Berrien.... 100.......... Blue Ridge....... Fannin........ 1.......
Avalon..........Franklin.	100......... Blue Springs....Gordon...................
Averett.........Decatur....	200 ........ Bluffton.........Clay......	200	1 ....
Ayersville......Hab’sham	75	1.... Boggy............Richm’d..	200	1 ....
Azalia..........Scriven....	300 ........ Bold Spring...... Franklin.	300	1 ....
Babb............Henry.....	50........ Bolingbroke......Monroe ...	50	2	2
Baconton........ Mitchell...	200	1	. Bolton......... Fulton....	200	1 ....
Bailey’s Mills... Camden ...	200	1 ... Bond’s Mill......Twiggs....	125	3 ....
Bainbridge......Decatur...	1500	3	. Boston...........Thomas...	450	1	1
Bairdstown......Ogleth’pe.	250	1	. Bottsford........Sumter ...	100	1.......
Baker...........Hall......	150......... Bowdon...........Carroll.... 350	3 ....
Baker’s Ferry... Elbert............ 3.... Bowdre...........Hall......	50.......
Ball Ground.....Cherokee..	250	2	. Bowersville......Hart......	200	1 ....
Balloon. .......Coffee................... Bowman........... Elbert...	300	4 ......
Banksville......Banks.....	150	2	1	BoxSpring...Talbot....	150	1.....
Banning ........Carroll....	300	1 ... Braganza.........Ware....................
Barber’s Creek.. Jackson ...	200	1	. Branchville......Laurens...	150........
Barnesville ....Pike...... 2000	5	. Brantley.........Marion ...	300	......
Barnett.........Warren ...	175	2	. Brass............Towns.....	300	.......
Barrettsville...Dawson....	500	2	......	Brasstown...Towns..___	100........
Table No. I.—Continued.
uii Ci	GO C3
S ©	°
O Q ® o	Oq ® O
TOWN. COUNTY. g g£ ° g TOWN. COUNTY. g g£ ® |
•2	s	e<!	.2	s
s	Z	“ _	03	X —•
I—<	Xi T?	•—*	-O u
g	«	g®	g	”5	SJ?
g< g
Ph	Eh	S	Ph	H
Bremen......... Haralson.	200	2 ... Cason........... Wilcox...	300	1	..
Brentwood...... Wayne....	500 ........ Cass Station....Bartow....	75	1	1
Brierville..... Floyd.....	250 ........ Cassandra....... Walker .................
Bright Star.... Douglas..	450 ........ Cassville....... Bartow	...	150	2	..
Brinsonville... Burke................... Cat Creek.......Lowndes .	100.........
Broad.......... Wilkes............. .... Cataula.........Harris....	150	1	..
Brookfield.....Berrien ...	250 ........ Cave Spring.....Floyd..... 1000	4	2
Brook’s Station. Fayette...	150	4 ... Cawthon......... Hancock.	250	1	..
Brown’s Bridge. Forsyth. ..	300	1 ... Cedar Grove.....Walker... 200	2 ..
Brown’s Cros’ng Baldwin................. Cedar Hill......Gwinnett	125.........
Brownsville....Paulding..	50	1 ... Cedar Ridge.....Whitfield	150.........
Brunswick......Glynn........ 4000	6 ... Cedar Springs...	Early...	50	1	.
Brusky.........Spalding..	100	1.... Cedartown.......Polk...... 2000	8	1
Bryan.......... Bryan.......	50	..... Centreville.....Gwinnett	2Oo	1....
Buchanan ...... Haralson.. 300	2 ... Centreville Box Walton ...	20o	1 ..
Buck Creek.....Seri ven ...	300	2 ... Ceres...........Crawford. 125............
Buck Hpad...... Morgan... 400	1 ... Chalybeate Spgs Meriw’her 200	1 ..
BuenaVista..... Marion....	600	3	1 Chamblee.......DeKalb... 60.............
Buford.........Gwinnett	600 ........ Chapel Hill.....Douglas........ 	2...
Bullards.......Twivgs.......	100	3 ... Chastain........Colquitt...	500 ..... ...
Bulo...........Cherokee..	200........ Chattooga ville... Chattooga	75	1 ..
Buncombe.......Dawson ...	100......... Chauncey........Dodge.....	350	1 ..
Buren.......... Union.....	600 ........ Cheap...........Banks.....	100.........
Burton.........Rabun........	100........ Cheevertown .... Baker....	100	2 ..
Butler......... Taylor......	800	5 ... Cheeves.........Carroll...	300	1 ..
Buzzard Roost.. Twiggs......	100	1	1 Cherokee Mills.. Cherokee.. 200	3 ..
Byromville .... Dooly.......	100	2	1 Cherry Log.....Gilmer....	300 ........
Byron.......... Jackson...	200	4 ... 'Chestatee......Forsyth...	500 ........
Cabaniss.......Mouroe ...	100	1 ... 'Chestnut Flat... Walker....	150......
Cairo..........Thomas...	400	3 ... Chestnut Gap... Fannin....	250 ........
Calhoun........Gordon ...	550	3 ... China Hill......Telfair...	250	......
Calvary........Decatur...	100	1.... Chipley.........Harris....	300	1	....
Camak.......... Warren ...	75	1.... Choestoe........ Union............. 1	..
Cameron........Scriven......... 2....... Chulio.......... Floyd....	300	1	...
Camil'a........ Mitchell... 1000	4	1 Church Hill ...Marion............. 2	....
Camp Creek.....Dooly.................... Cicada..........Cobb......	100.........
Campagne.......Towns.................... Cisco...........Murray ... 200...........
Campbellton....Campbell.	100	1.... Clarkesville....Hab’sham	500	4	....
Camps..........Telfair....	400	..... Clark’s Mill....Crawford..	200	1	..
Candler........ Hall......	150	..... Clarkston....... DeKalb....... 1	1
Canoochee...... Emanuel.. 75	1	1 Claude......... Wash’ton 200 ...........
Canton ........Cherokee.. 500	5 ... Clay Hill....... Lincoln ...	100	2 ..
Captolo........Scriven.... 100	1.... Clayton.........Rabun.....	400 ........
Cardville......Jones........... 1.......' Clayville...... Telfair.................
Carlisle.......Gilmer.....	500 ........i Clemeth........ Union....	200 ........
Carnesville....Franklin..	250	4 ...... Cleveland.... White....	300	4	..
Carrollton ....Carroll.... 1750	5	2 Climax.........Decatur... 50............
Carr’s Station... Hancock..	300	1 ... Clinton........... Jones..	200	6	1
Carsonville..Taylor.......	200	1 ... Cloptou.......... Putnam...	200	2	.
Cartecay.....Gilmer.............. 2.... Cloverdale.....Dade.........	50	1...
Carter.......Murray...	500	1 ... Clyattville....Lowndes..................
Cartersville. Bartow...... 3000	9 ... Coal Mountain..	Forsyth....	300	1 ..
Table No. I.—Continued.
2	«	“ cs
°
Jj °	?.§ _S*o
& ”, ~ J3	® £ ~ «
TOWN. COUNTY. g g£©i TOWN. COUNTY. g ©|
•2	©	E<;	.2
£
g	M	g kg	g'gg0
Q.	■“	Jn S	a.	4=	R SJ
o	□	o	o
H	H	<<	Pn	Eh	S
Cobbyille......Te.fair....	50......... Cutcane........Fannin.... 500 ..........
Cochran .......Pulaski.... 1300	6	3 Cuthbert...... Randolph 2500	9 ....
Cohutta........ Whitfield f50........... Dahlonega......Lumpkin. 1000	3 ....
Cohutta Springs Murray ...	150	2 ... Dale’s Mills... Wayne.....	150........
Cold Water..... Elbert.../.	100	1 ... Dallas......... Paulding..	600	4	1
Cole City...... Dade.................... Dalton......... Whitfield	3000	6	2
Coleman’s Dep’t Randolph 250	1 ... Damascus.......Early......	150	2 ....
Collier........Monroe...	75	1 ... Danburgh....... Wilkes....	300	1	.
Colquitt....... Miller....	300	5 ... Daniels Mills.. Douglas... 225 .........
Columbus....... Muscogee. 20000	30	4	Danielsville. Madison...	300	2 ....
Commissioner... Wilkinson 40	1	. Darien.........McIntosh......	2.....
Commodore...... Tattnall................ Darlot.........Liberty.... 70	3 ....
Concord........ Pike......	300	1	. DaviB’ MUIb.... Wilcox....	100........
Concordia...... Elbert....	300 ....... Davisborough ... Wash’ton 300	2 ....
Condor......... Laurens... 150.......... Dawnville...... Whitfield ........ 1	..
Conley.........Clayton.................. Dawson.........Terrell.... 2000	4 ....
Conyers........Rockdale.. 1700	3 .... Dawsonville...Dawson.... 200	1 ....
Coogle’s Mill..Macon......	150	2 ... Decatur........DeKalb ...	900	4 ....
Cookstown...... Wilcox.................. Delmar......... Lowndes 300 ....... ....
Cooksville.....Heard.............. 1	.. DeSoto......... Floyd..... 1200	1 ....
Cool Spring....Wilkinson 150	3 ... Devereaux Sta’n Hancock.. 200	2 ....
Coosa..........Floyd......	100	1 ... Dewyrose.......Elbert.....	100........
Coosa Creek.... Union.....	450 ....... Diamond.......... Gilmer..	200 .......
Coosaville..... Floyd............. 1	1 Dillon .......Walker...................
Coosawattee....Gordon....	150	1	.. DirtTown.......Chattooga	150	2	1
Copeland.......Dodge.............. 2.... Dixie..........Brooks.....	300	3 ....
Cora...........Newton...	50	1	. Dobog.......... McIntosh	300	1 ...
Corinth........Heard.......... 1	.. Doctor	Town.....Wayne.....	100........
Cornucopia..... Jones..... ....... 2.... Don............Harris.....	100........
Cottage Mills.... Cha’h’hee	200	2 ... Doraville......DeKalb......... 2........
County Line Carroll  86................. Dorchester.....Liberty.... 200 .........
Cove City......Whitfield. 100........... Dorminey’s M’ls Irwin  100..............
Covena......... Emanuel..	200 ........ Double Bra’ches Lincoln ...	225	5	.
Covington......Newton...	2000	3	1 Dowdy.........Madison...	275	1	..
Crackling...... Banks.....	75	2 ... Dougherty......Dawson....	150	3	..
Craftsviile....Elbert.....	150........ Douglas........ Coffee....	50	1.....
Crane Eater....Gordon...	125	1.... Douglasville...Douglas...	.1000	3	3
Crawfish Spr’gs.. Walker....	100	2 .... Dove’s Creek...Elbert...................
Crawford.......Ogleth’ pe	400	3 ... Draketown...... Haralson........	3....
Crawfordville.... Taliaferro	600	3 .... Drayton........Dooly......	200 .......
Crayton..........Fannin...	150........ Drewryville.... Spalding..	150	1.....
Creswell.......Spalding .	325	1	. Drone..........Burke......	100	2 ....
Cromois........Franklin......	1 ... Dry Branch.....Bibb.......	100........
Cross Keys.....DeKalb...	40	1	_ Dublin.........Laurens...	620	3	2
Crystal’Spring... Floyd...	125	2	. Dubois.........Dodge......	300	......
Cuba...........Early......	100........ Duck Creek..... Walker.... 300 .........
Culloden....... Monroe... 300	2	1 Duffle........ Wilcox ...	200 .......
Culverton...... Hancock .	125	1.... Duluth.........Gwinnett. 500	2 ....
Cumberland.....Camden... 40............. Dunn...........Murray...................
Cumming........ Forsyth...	350	6	. Dunwoody....... DeKalb....	40	1......
Curtis......... Carroll...	175	5	. DuPont.........Clinch.....	600	1	.
Cut seta.......Cha’h’hfe	300	4	. Durden.........Emanuel..	50j	3......
Table No. I.—Continued.
cn c3	qc oj
Ja o	^*0
. -q
TOWN. COUNTY. g 8£ => $ TOWN. COUNTY. g Sg 0 8
.2 s	2 <	.2	=s	“ <1
2	® .	2	«	•
•	t!	'O
'& 3	a	a
00"^	o	o
ft ft	»3	ft	ft	3
Dyke’s Store...Floyd......	100	1.... Field’s X Ronds Milton.....	300........
Eagle Cliff.... Walker.... 100	2.... Fillmore.......Whitfield 500	3 ...
Eagle Grove....Hart.......	150	1.... Fish...........Polk........	150........
East Point.....Fu’.ton.	...	300	4 ... Fitts.......... Carroll....	300........
EastanoLee..... Franklin .	100...... Five Points.... Jones......	300	2 ...
Eastman........ Dodge.....	600	5	1 Flat Branch...Gilmer......	200 .......
Eatonton.......Putnam ... 1000	5	1 Flatt Creek...Fayette ...	300	2 ...
Ebenezer.......Dooly......	100...... Flat Shoals....Meriw’her 100	1 ...
Echeconnee..... Houston ..	150......... Fl’tW’d Acdmy	Elbert...	100	1....
Eden..........  Effingh’m	200	1 ... F eming........Liberty....	250	1..,.
Edgewood.......DeKalb ...	1000	2 ... Flint..........Mitchell...	100	1 ...
Edwardsville... .. Fulton ....	250	1 ... Florence....... Stewart ...	200	3 ...
Egypt .........Effingh’m 200	1	1 Flowery Branch Hall.........	400	2 ...
Elberton.......Elbert..... 1400	7	1 Floyd Springs... Floyd........... 1	1
Ellaville......Schley.....	200	2	. Folks..........Calhoun...	250	1 ...
Ellerslie......Harris.....	100	1.... Folkston.......Cheriton..	150	1....
Ellijay........Gilmer....	300	2	. Ford’sStore....IHart........	300 ......
Eliza..........Tattnall ..	200 ........ Forrest, Hall..Bnrke.......	100	3 ...
Emily..........Carroll....	400 ..... Forestville....Floyd.......	700	1 ...
Enecks.........Scriven.... 300 ......... Forsyth........Monroe... 1500	3 ...
Enigma......... Berrien... 150.......... Ft. Buffington.. Cherokee......	1....
Enon Grove..... Heard.....	250 ..... Fort Gaines....Clay........ 1200	4 ...
Enterprise..... Lee.......	100	1.... Fort Lamar..... Madison....... 1	1
Enville........ Wayne.....	500	1 ... Fort Mountain.. Murray ...	100........
Ephesus........ Douglas. ..	80.......... Fort Valley.... Houston	..	2000	6	2
Erastus........ Banks.....	75	2 ... Fortner........ Emanuel. 75	2 ...
Eriu...........Pike.......	150	2 .... Foster’s Store... Chattooga	140.......
Esom Hill......Polk.......	200	1 .... Fouche......... Floyd.. 1000...........
Etowah.........Floyd......	500	1 .... Fowlstown......Decatur.................
Eubanks........ Columbia	500	1 .... Franklin....... Heard......	300	......
Eudora.........Jasper.....	50.......... Frankville.....Monroe ...	150.....
Euharlee ...... Bartow....	225	1	1 Frazier......... Pulaski....	50.....
Eula...........Jasper  5Oo.............. Freemansville .. Milton........ 1 
Eureka.........Dooly......	100	2 .... Frick’s Gap.... Walker....	75.....
Eva............Telfair....	75.......... Friendship.....Sumter......	200	1 .
Evelyn.........Glynn...... 1500....... Gaddestown.......Union.......	300.....
Evens..........Columbia	40	2 .... Gainesville....Hall........ 2750	5	1
Everett’s Spi"gs Floyd  200 ............ Garden Valley... Macon......... 2 
Everett’s Stati’n Crawford.. 300 ....... Gari........... Wayne... 225 ..........
Evergreen .....Irwin......	300	1 ... Garfield.......Emanuel......... 2......
Excelsior...... Bulloch. ..	200	3 .. Garrant.........Coffee......	500........
Exeter.........Pierce.....	250 ..... Geneva.........Talbot......	450	2 ...
Fair Mount.....Gurdon....	100....... Georgetown....... Quitman..	300 .......
Fair Play...... Morgan ...	200 ......... Gerber.........Walker....	200 .......
Fairburn ......Campbell.	700	6 ... Gibson.........Glascock........	3....
Faircloth...... Mitchell.	500	1 ... Gilgal.........Scriven....	200........
Fancy Bluff. ... Glynn....... 1......... Gillsville..... Banks......	150........
Fayetteville...Fayette ...	250	4	1 Girard.......... Burke.......	200	1 ...
Feronia........Coffee................... Gladesville....Jasper..................
Ferry..........Floyd......	200 ..... Glenalta.......Marion......	125........
Fido...........Bryan......	100	1.... Glenmore ......Ware........	200........
Table No. I.—Continued.
<n of	tn cj
S 0	S 0
73	<4-4 03
0	<D <4-4	O	® <4-<
’.Edo	Liao
I "	a	| & O
TOWN. COUNTY. g aS ° “ TOWN. COUNTY. g g£ ® S
.2	s	g<l	.2	g<1
«	*	*	*	Sv
g.	«	g,	s
o	O	o	o
Ph	H	S	Ph	H
Goggansville. ... Monr e ...	100....... Harville........Bulloch ...	400 .......
Goldsborough.... Pulaski...	150	1.... Haslam..........Wayne.....	200 .......
Goloid.........Scriven ...	300 ....... Hassler’s Mill... Murray ...	300 .......
Good Hope ..... Walton... 150	1.... Hat............. Irwin.... 150..........
Gordon.........Wilki’son	450	4 ... Hatcher’s Stat’n	Quitman .	150	2	.
Gordon Springs. Whitfield	200 ........ Hawkinsville....	Pulaski...	2500	8	5
Goshen.........Lincoln ..	100	1... Hayneville......Houston..	150	3	.
Grace..........Lumpkin 200 ............ Hazlehurst......Appling... 300	3 ...
Graham......... Appling ..	250 ....... Head’s Ferry....White.....	250 ........
Grangersville.... Macon..	100	3 --- Heard...........Butts.....	150........
Grantville ....Coweta ...	700	3 ... Heardmont....... Elbert...	200 .......
Grapevine......Gwinnett 300 ........... Heath...........Burke.....	200	1 ...
Graysville.....Catoosa ...	450 ...... Hempstead ......Colquitt... 200 ........
Greene.........Bulloch...	100	....	. Henderson....... Houston ..	200	1	.
Grbene Hill....Stewart... 300	4 ... Hephzibah.......Richm’d... 300	2 ...
Green’s Cut.... Burke....	200	1 ... Hermitage.......Floyd.....	250	1	.
Greensborough. Green..... 1600	5 ... Herndon.........Burke............. 4	2
Greenville.....Meriw’her 500	3 ... Hiawassee.......Towns.....	300	2 ...
Greshamville .. Green....	200	3 ... Hickory Flat... Cherokee 300	1 ...
Griffin........Spalding . 4000	12	2 Hickory Grove.. Montg’ry...............
Griswoldville.... Jones..	100........ High Falls......Monroe...	200	1	.
Grove Level....Banks.....	75	1.... High Shoals.. .. Walton ...	650	5	1
Grovetown...... Columbia 350	2	1 Hightower...... Forsyth...	100	1....
Guess..........Henry........... 1...... Hillsborough.... Jasper...	300	1 ...
Gully Branch... Coflee...	200	..... Hinely .........Effingh’m	150	1....
Gum Creek......Dooly.....	150	1.... Hinesville......Liberty..........	1....
Gum Spring_____ Bartow... 100.......... Hix.............Madison.. 150 .............
Guyton......... Effingh’m 250	3 ... Hoboken ........ Pierce...	200 .......
Hack Branch.... Montg’ry. 125..... .... Hogansville.....Troup.....	500	5 ...
Haddock Stat’n Jones ....... 75	1.... Hoggard’s Mill.. Baker....	150	2 ...
Hahira.........Lowndes.. 300 .......... Holcombe .......Burke...................
Haides.........Scriven ...	300	1 ... Holland’s Mill.. Carroll.......... 1....
Haines.........Lowndes . 100........... Holland’s Store Chattooga 200 ..........
Halcyondale Scriven... 500 3   Hollingsworth.. Banks  150.......................
Hall’s Mill....Bartow ...	100	..... Hollonville....... Pike...	250	1	.
Halloca........Cha’h’hee	75........ Holly Creek..... Murray ...	200	1	.
Hamilton.......Harris....	500	3	1	Holly Springs...	Cherokee	75	1....
Hammond........Fulton....	200	1 ... Hollywood.......Richm’d—................
Hammon’s Mill Floyd.................... Holmesville.....Appling...	100.......
Hamp Branch... Scriven....	150....... Holton..........Bibb......	150	1....
Hampton........ Henry....	700	2 ... Homer...........Banks.....	200	2 ...
Hannahatchee.. Stewart...	175	2 ... Homerville......Clinch....	450	1 ...
Hapeville......Fulton....	100	2 ... Hooper..........Haralson................
Haralson.......Coweta... 125	2 ___ Hopewell........Colquitt...1 150	1 ...
Hard Cash .....Elbert....	50	1.... Horace..........Emanuel.................
Hardaway.......Dough’rty .............. Horn’s X Roads Miller.....	150	1....
Hargett........Harris....	200	2	. Hot House.......Fannin	...	150........
Harlem.........Columbia	350	5	1	House Creek..Wilcox...	250	2 ...
Harmony........Putnam..........	2.... Houston.........Heard.....	100	3 ...
Harmony Grove Jackson...	500	4	2	Howard.......Taylor.......... 2......
Hartwell.......LHart.......	600	5	. Howell’s Mills.. Fulton.................
Table No. I.—Continued.
tn	d	tn	cd
§	e>	g	o
© O	2*5	O-g	©'g
£ ”® « c	g ’® +a a
TOWN. COUNTY g	| TOWN. COUNTY g g£ ° I
3 ZK	-2 2C
* *
g. 3	3 3 8~
T o	©^	00©*5
p H	3	Ph E-i S
Huff...........Gwinnett. 50............. Kiokee...........Columbia 150	1 ...
Hughes......... Murray ... 7g........... Kirkland......... Coffee..	150	2 ...
Hulmeville----- Elbert.....	150	1 ... Knoxville........ Crawford.. 250	3 ...
Hunt........... Towns................... LaFayette........ Walker.... 300	3 ...
Huntsville.....Paulding.. 75	1 ... LaGrange.........Troup ....	2700	6 ...
Ila............ Madison... 100.......... Lairdsborough .. Carroll..	150	3 ...
Indian Springs.. Butts.....	300	2 ... Lamar............ Sumter....	80	1....
Iric........... Bulloch...	100	1 ... Lamar’s Mill..... Upson.................
Iron Rock......Franklin..	125....... Langtry.......... Emanuel..	150........
Irvine......... Cobb.......	170....... Laston........... Bulloch... 400	1 ...
Irwinton....... Wilki’son	300	3	2 Lauren’s	Hill... Laurens...	100	1 ...
Irwinville..... Irwin................... Lavonia.......... Franklin.. 200	1 ...
Isabella ...... Worth......	150	1 ... Lawrenceville...	Gwinnett.	600	5	.
Ivanhoe........ Bulloch...	150....... Lawson........... Colquitt-	100	1...
Ivy Log........Union.............. 1	.. Lawtonville...... Burke...	300	1 ...
Jackson........ Butts......	600	3 ... Leary’s..........Calhoun...... 2........
Jacksonville...Telfair............ 1	.. Leathersville... Lincoln...	100	1....
Jacobs.........Berrien....	125	1	.. Ledbetter........ Baker...	75........
Jamaica........Glynn.......	100....... Leesburgh........ Lee.....	400	4 ...
James..........Jones.......	175....... Leliaton.........Coffee...	100	1....
Jamestown......Chath’che	500	2 ... Leno.............Hab’sham	300	1 ...
Jasper......... Pickens ...	325	1 ... Leo.............. White...	100	2 ...
Jay............Lumpkin.	250 ....... Leonard . ....... Bryan...	100	.....
Jefferson......Jackson...	800	4	2	Leverett.. Lincoln...	100	4	.
Jeffersonville.... Twiggs..	250	4	1	Lexington. Ogleth’pe	500	3	1
Jenkinsville...Pike........	100. Liberty Hill........... Pike....	200	1	.
Jerusalem......Pickens... 200	1 ... Lifsey’s Store.... Pike.................
Jesup..........Wayne... 700	2 ... Lily Pond........Gordon.... 200 ........
Jewell’s.......Hancock.........	3 ... Lime Branch.....Polk......	100	1 ...
Jewelville.....Banks.......	100....... Lincolnton....... Lincoln... 500	4 ...
Jimps..........Bulloch... 400 .......... Linton........... Hancock.. 150.........
Joel...........Carroll.... 100.......... Lithonia.........DeKalb... 500	2 ...
Johnston Stat’n Liberty ...	200 ...... (Little Creek....Haralson.. 350 ........
Johntown.......Dawson... 300 ........... Litt’e Row.......Gordon.... 100 ........
Jones’ Mills...Meriw’thr	200 ....... Littleville......Clayton...	100........
Jonesborough ... Clayton ...	1500	3 .... Livingston.......Floyd....	175........
Jordan’s Store... Pike.....	125	1.... Lizzie...........Cobb.....	50........
Joseph.........Fulton.... 150	3 ... (Locust Grove....Henry....	300	1 ...
Jug Tavern..... Walton....	150	2 .... (Lodi............Coweta...	100........
Juliette....... Monroe.... 300	1 ... Lodrick.......... Randolph 125	1 ...
Juniper........Marion......	75........ Loganville.......Walton.... 150	3 ...
Juno.............. Dawson ...	250 ...... Lombardy.........McDuffie .	250 .......
Kate...........Pierce......	150....... Long Branch......Tattnall... 100	2 ...
Kedron.........Coweta ...	250	1 ... Long Cane........Troup....	200	1 ...
Keith..........Catoosa...	130......... Long Pond........ Lowndes.	100.........
Kennady........ Bryan......	100....... Long Swamp....... Pickens...	100........
Kennesaw.......Cobb ........... 1....... Long View........Dodge....	200 ........
Key............Brooks......	200	2 ... Longstreet....... Pulaski.... 150	2 ...
Key ton........Calhoun... 150	1.... Lost Mountain.. Cobb......	400 ........
Kimbell........Butts.................... Lothair..........M’tgomry 100...........
Kingsborough... Harris.....	150	1.... Loudsville....... White...	300	1 ...
Kingston ......Bartow ... 500	1 ... Loughridge.......Murray ... 150.........
Table No. I.—Continued.
<n	cj	on cj
§ o	go
0’5	o-S
a c	55 ■” ’S c
TOWN. COUNTY g a£°i TOWN. COUNTY g gg’oS
=>	£ <	T!	g <1
3	5	-	5
a 5 8j •	ft S
O	O	O	O
ft	ft	3	ft	ft	£
Louisville.....Jefferson.. 850	5	1 Merrell.......Heard......	75.........
Lovejoy’s Stat’n Clayton.... 100	2	1 Mesena........Warren... 250 ....... ...
Lovelace.......Troup......	150	1..... Mica...........Pickens... 75............
Lowell......... Carroll...	100........ Middle Ground Scriven.... 500 ..........
Ludville....... Pickens...	150	1 .... Middle River... Franklin..	125	1 ...
Lula........... Hall......	75......... Midville.......Burke......	150	3 ...
Lulaton........Wayne......	150........ Milford........Baker......	100	1....
Lumber City....Telfair....	200	2 .... Mill Haven.....Scriven ...	150......
Lumpkin........Stewart...	1000	3	.. Mill Ray....... Bulloch...	200	3 ...
Luther......... Warren...	125	2	.......	Milledgeville. Baldwin..	4000	9	6
Luthersville...Meriwth’r	300	2	.. Millen......... Scriven....	350	2 ...
McArthur.......M’tgom’y	300 ........ Milltown.......Berrien....	175	1....
McBean Depot.. Riclim’nd 250	2 .... Milner.........Pike.......	600	3 ...
McConnell......Cherokee.	300 ........ Milner’s Store... Fayette ...	150	1....
McDade......... Richm’nd 100	1 .... Mimosa.........Walker.... 125	3 ...
McDonald....... Thomas...	200	2 .... Mineral Springs Pickens ...	200	.....
McDonald’s Mill Coffee....	100	1..... Minton.........Worth......	110......
McDonough......Henry......	500	3 .... Mitchellton....Scriven.... 75	4 ...
McIntosh....... Liberty... 150	4 .... Mobley Pond....Scriven.... 150	4 ...
McLellan’s Mills Worth....	100	1..... Monroe.........Walton... 900	4 ...
McLemore....... Walker.................. Monteith........ Chatham.. 100..........
McRae..........Telfair....	400	1 .... Montevideo ....Hart.......	75.........
McVille........Telfair............ 1.... Montezuma......Macon......... 2	1
Mableton.......Cobb.......	50	1..... Monticello.....Jasper.....	520	4 ...
Macedonia......Cherokee..	200	1	. Moore’s Mills....	Cherokee.	100......
Macon.......... Bibb...... 23000	24	19	Morgan.......Calhoun ..	230	5 ...
Madison........ Morgan...	2600	5	3	Morganton....Fannin....	150	1....
Magdalena...... Meriwth’r 150........... Morganville....Dade.......	150........
Malden Branch.. Bryan.....	150........	Morris Station... Quitman.. 100	1....
Mallorysville.. Wilkes....	175	1..... Morrow’s Stati’n Clayton...	250	1 ...
Marcus.........Jackson .	100........ Morven.........Brooks........ 2.........
Marietta.......Cobb....... 3000	10	1 Mossy Creek... White.....	600	2 ...
Marlow’........Effingh’m	150	1..... Moultrie.......Colquitt...	300 .......
Marshallville.. Macon.....	400	6 .... Mount Airy.....Habr’ham	250	2 ...
Martin.........Franklin..	100	1..... Mount Pleasant Wayne......	150......
Marysville..... Johnson...	150	1..... Mount Vernon.. M’tgomry	150	2 ...
Math...........Emanuel..	75......... Mount Zion.....Carroll....... 5.........
Mattock........Tattnall...	100	1..... Mountain Hill.. Harris....... 3.........
Mauda.......... Hancock..	200 ........ Mountain Scene Towns______	100......
Mauldin’s Mills Hall........	100........ Mountaintown .. Gilmer....	250	.....
Maxey’s........Ogleth’pe. 300	5 .... Mountville.....Troup......	300	3 ...
Mayfield.......Hancock..	200	2	. Moxley.........Jefferson .	300	1 ...
Maynard........ Monroe ...	150	1.... Mulberry.......Jackson....	300	1 ...
Maysville......Jackson.... 300	1	... Mulberry Grove Harris..	125	1....
Mazeppa........Milton.....	150	2	. Mulville.......Chath’che.....	1....
Mechanicsville.. Jasper...	75......... Munnerlyn......Burke......	150	1....
Meigs..........Thomas...	200	1 .... Murry’sXRoads Schley......	100	1 ...
Melrose........Echols.....	300	...... Myra........... Appling... 100	1....
Melville.......Chattooga 150	1..... Myrtle.........Houston ..	100........
Menlo..........Chattooga	125........ Nacoochee......White......	300	2....
Meriwether.....Baldwin..] 200]	3..... Nahunta........Wayne....	150........
Table No. I.—Continued.
cn cj	cn <3
°«Jo	°35o
•	l^~-°
TOWN. COUNTY. g s£ ® I TOWN. COUNTY. g ° |
1	| |2	|	5	Id
g.	3	s.	3
O	O	o	o
Cl,	H	Pl,	H
Nails Creek.... Banks.....	150........ Ousley..........Lowndes..	250 .... ....
Nannie.........Floyd......	125	1 .... Oval............Paulding..	40...
Naomi..........Walker....	75......... Overton.........Elbert.....	50	1	....
Napoleon....... Union.....	100........ Owen’s Ferry...	Camden...	150...
Nashville...... Berrien...	250	1	1 Owensbyville....	Heard....	150...
Naylor......... Lowndes..	600	1 .... Oxford..........Newton...	800	1
Nebo...........Paulding..	200 ........ Palmetto........Campbell.	500	7
Nellwood....... Bulloch...	250	1 .... Panban.......... Warren...	150	J
Newbridge......Lumpkin.	500 ........ Panola..........DeKalb...	300	1
New Hope.......Paulding..	100........ Pantherville....DeKalb ...	75	J
New Market.....Monroe....	100	1.... Paper...........Clarke.....	250	.
New Prospect... Forsyth...	250	1 .... Parker’s Store...	Hart.....	175	2
New Providence Wilki’son.	75	1 .... Paschal.........Talbot.....	100....
New Switzerl’nd Hab’sham	100........ Pateville.......Dooly......	100....
Newborn........... Newton...	150	1.....!	Paxton.......Thomas... 100.......
Newnan.........Coweta..... 2500	8	2	Pay Up.......Hart.......	200	1
Newton.........Baker......	300	2	. Peachstone Sh’ls Henry.....	100	c
Newton Factory Newton ...	100 ....... Pearson.........Coffee.....	300 ......
Nicho'ls.......Coffee.....	175	1. Peck...............Worth......	175....
Nicholson......Jackson...	160	1.... Pedrick.........Brooks..................
Nickajack...... Walker....	100.......... Peeksville.... Henry.....	80	2
Nickviile...... Elbert....	75	1.... Pelham.......... Mitchell...	300	1
Nielly.........Telfair....	75......... Pendarvis....... Wayne.....	300 ......
Norcross.......Gwinnett	700	6 ... Penfield........Greene....	465	4
Norton.........Whitfield 75	1	1 Pennington.....Morgan ...	75....
Norwood........Warren ...	200	2 ... Perkins’ Junc’n	Burke....	200	4
O’Neal’s Mills... Troup...	250	1 ... Perry...........Houston ..	1200	I ;
Oak Bower......Hart.......	150........ Perry’s Mills...Tattnall...	200	J
Oak Ridge...... Meri’ther.	75......... Persimmon.......Rabun......	300	.
Oakland........ Meri’ther. 500 ......... Phideita........ Banks..............
Oakley......... Cobb......	50......... Phillips’ Mill..Coffee.....	75........
Obe............Colquitt...	100........ Philomath.......Ogleth’pe.	150	2
Ochlochnee.....Thomas ...	200	2 ... Picciola........ Laurens...	100...
Ocilla.........Irwin.................... Pickren.........Coffee..............
Ocmulgee....... Monroe.... 500	1 ... Pierceville..... Fannin.... 400 ........
Oconee.........Wash’ton. 250	1 ... ,Pike...........Montg’ry. 75....	__
Octavia........Cobb.......	75	1______ Pine	Grove....Appling...	200 ........
Ogeechee.......Scriven....	100	2	. Pine	Log......Bartow.....	200	2 ..
Oglethorpe..... Macon.....	300	1	. Pine	Ridge....Twiggs.....	50	2
Ohoopee........Tattnall...	150........ Pinebloom.......Coffee.....	100........
Okapilco....... Brooks....	200 ........ Pineville....... Marion	........ 2	....
Okefinokee ....Clinch.....	50......... Pistol.......... Wilkes....	150	2
Oldham......... Ware......	50.... .... Plains of Dura.. Sumter ...	100........
Oliver......... Scriven....	150	1.... Plainville...... Gordon	...	150	2	....
Oostanaula.....Gordon....	75........... Planter.......Madison...	300 .... ...
Ophir.......... Cherokee.. 150	1 ... Pleasant Grove.. Forsyth... 200	3 ..
Orange.........Cherokee .......... 1	.. Pleasant Hill...Talbot.....	200	2 ..
Orchard Hill.... Spalding..	100	2 ... Pleasant Retreat White.....	75........
Orsman.........Floyd......	200	...... Plowshare.......Carroll....	125........
Osborn.........Towns......	200	. .... Poindexter......Schley.....	100........
Osceola........Oconee.....	100	1	1 [Point Peter...Ogleth’pe. 250	1 ..
Table No. I.—Continued.
oo	cs	tn oj
§	o	So
°.2 3*8	° 2 .So
.
TOWN. COUNTY g g£ ° " TOWN. COUNTY g a£
•2 o	‘	-2 3^ ZH
ft 3?	ft 3 S£
o O	o o
ft H	ft H
Polkville...... Hall......	50........ Rehose..........Haralson..	500 .......
Pond Fork......Jackson	..	200	2 ... Resaca..........Gordon...	200	2 ......
Pond Spring....Walker ...	120	2 ... Reuben..........Chattooga...............
Pond Town......Miller.....	100........ Rex............. Clayton...	400	2....
Poore’s Mill...Colquitt.. 300 .......... Reynolds........ Taylor....	300	4 ...
Pope...........Jefferson..	150	5 ... Riceborougb.....Liberty...	500 .......
Poplar Hill Telfair..................... Richland........ Stewart... 300 5 
Poplar Spring... Haralson.. 150 1   Riddleville..........Wash’ton    1 
Porter Springs.. Lumpkin. 300 .......... Ringgold........Catoosa... 500	8 ...
Postell........Fannin ...	125	1 ... Rising Fawn.....Dade.......	500	2 ...
Pots Mountain.. Dawson ...	200	2 ... Rivertown.......Campbell. 300 ..........
Poulan.........Worth......	125	1	.. Rives...........Dough’ty.	200	1	.
Powder Springs Cobb.......	400	5	.. Rock	Fence...Elbert......... 1	.
Powellville....Coweta	...	150	1.... Rock	Mart____Polk....... 1000	5	1
Powelton ......Hancock	.	140	2	.. Rock	Pond....Decatur........ 1	.
Powers.........Terrell......... 2....... Rock Spring..... Walker... 150	2 ...
Poweraville....Houston ..	200 ...... Rockalo.........Heard......	275	1 ...
Prattsburg.....Talbot.....	100	1.... Rockpile........Dawson...	150	1....
Preston........Webster	..	250	3	.. Rocky Creek.....Gordon ...	150	1....
Price...... ...Hall............... 4.... Rocky Ford......Scriven ...	125	1	.
Pringle........ Wash’ton. 175........... Rocky Mount... Meriw’her 300	2 ...
Prior’s Station.. Polk.................. Rogers..........Burke  150..............
Providence  Sumter... 175............... Rollins.........Paulding. 100...........
Pruit..........Banks.......	100........ Rome............Floyd...... 9000	12	7
Puckett Station Coweta ...	200	3 ... Roopville....... Carroll...	300	3 ...
Pulltight......Decatur...	150........ Roscoe..........Coweta....	200	1	.
Pumpkin........Paulding..	250 ........ Roswell.........Cobb....... 1200	4	1
Putnam.........Marion......	150	1.... Round Oak.......Jones......	100.......
Pye............Wayne....	125......... Round Tree......Emanuel..	300 ..........
Quantock.......Scriven ...	100........ Rouse...........Worth......	125.......
Quebec.........Union.......	150........ Ray.............Gilmer....	150.......
Quitman........Brooks...... 2000	4 ... Royston ........Franklin..	300	1 ...
Rabun Gap......Rabun.......	300	...... Ruckersville....Elbert.....	100	1....
Raccoon Mills... Chattooga	232 ........ Rural Vale......Whitfield	100.......
Racepond.......Charlton..	100........ Russellville	...... Monroe...	100	1....
Ramsey.........Murray...	150	1.... Ruth............Greene.....	150	1....
Randel.........Colquitt................. Rutledge........Morgan ...	450	3	1
Ray’s Mill.....Berrien...	150	1.... Ryonville.......Liberty...	100.......
Raytown........Taliaferro......	1	1 St. Marks......Meriw’h’r 300	2 ......
Red Bluff......M’tgomry 150............. St. Mary’s .....Camden... 1000	2 ...
Red Bud........Gordon... 75............. St. Simon’s M’ls Glynn.....	800 .......
Red Clay.......Whitfield ...... 2	2 Sallacoa.......Cherokee.. 250	1 ...
Red Hill.......Franklin.. 150........... Salt Springs.... Douglas.... 300	1 ...
Red Oak........Campbell 300	1 ... Sand Hill.......Carroll.... 125	2 ...
Redan...........DeKalb... 75............ Sand Town.......Campbell 100............
Reddish........ Wayne................... Sandersville ....Wash’ton 1500	5	3
Reed Creek..... Hart.......	75.......... Sandy Cross.....Ogleth’pe....	1....
Reedy Springs .. Laurens.... 300 ....... Sandy Ridge.....Henry......	200 ........
Reese..........Morgan... 200	1 ... Santa Luca......Gilmer.....	200 .......
Regnant........Johnson.........	1.... Sardis..........Burke......	300	2 ...
Reedsville.....Tattnall... 175	2 ... Sasser.......... Terrell......... 2|....
Table No. I.—Continued.
tn	aS	<n	rf
§ o	~ e>
c (a 2 c	o ’6 ®
$5 >> S	al c
TOWN COUNTY. ©	o 2 TOWN. COUNTY. © s£ © S
.2	g	.2	s	g<f
«	5	3-d	3	*	3-d
g.	3	£	3	Ss
Ph	H	3	Ph	Eh	3
Satilla Bluff..Camden................... Stamp Creek.....Bartow ...	250	4 ...
Savannah.......Chatham.. 41000	35	14 Stansell.....Elbert.....	50	1....
Saw Dust....... Columbia 200	2 ... ;Stapleton.....Jefferson .	200	2 ...
Saw Mill.......Chattooga 125 ........... Stark.......... Butts.....	225	1 ...
Scarborough....Scriven ...	200	3 ... State Line.....Heard......	250 ........
Scarlett.......Camden... 225	1 ... (Statenville...Echols.....	300	1 ...
Schlattervilie.... Pierce.	100 ........ Statesborough... Bulloch... 150	1....
Scova..........Berrien ...	110	1.... Steam Mill.....Decatur... 150	2 ...
Screven .........Wayne...	300 ........ Stearnesville..Pike.......	125	1....
Seed............ Hab’sham	100......... Stearns........Pickens...	150........
Sellers........Appling.................. Stella.........Lowndes.. 50.............
Senoia.........Coweta ...	900	4	1 Btellaville...Jefferson.. 100	6 ...
Settendown..... Forsyth... 125 ......... Stephens.......Ogleth’pe 225	2 ...
8eward ........Montg’ry.	75	1	.. Stephensville ... Wilki’son	125	1	1
Shady Dale.....Jasper....	300	4	.. Sterling Station Glynn....	100.........
Shady Grove....Carroll ...	125 ........ Stevens’ Pottery Baldwin ..	150	2 ...
Sharon..........Taliaferro	400	1	.. Stewart’s Mills.. Schley..	50.........
Sharp Top......Cherokee 100............. Stilesborough ... Bartow ...	400	2 ...
Sharpsburg.....Coweta	...	300	2 ... Sterling.......Montg’ry	150........
Sheltonville...Forsyth...	150	2 ... Stockbridge....Henry......	250	3	.
Shepherd.......Coffee...	100........ Stockton.......Clinch.....	300........
Shiloh.........Harris....	150	2 ... Stone Mountain DeKalb ...	900	4	1
Shoals.........Warren................... Stuckey........Montg’ry. 25	1 ...
Shoulder.......Hancock .	75......... Subligna.......Chattooga	150	1	1
Silcam ........Greene ...	150	1.... Sugar Hill.....Hall.......	100	2	.
Silver Creek...Floyd........... 2....... Sugar Valley...Gordon ...	415	2....
Silver Shoal...Banks.................... Sumach.........Murray ...	60.........
Simsville...... Carroll..	100	2 ... Summerfield .... Bibb.....	300	2 ...
Skeinah........Fannin..., 200 ........... Summertown	... Emanuel   2 ....
Smarr’s Station Monroe...	200	1 ... Summerville....Chattooga	400	3	.
Smiley.........Liberty ...	100......... Sumner......... Worth.....	500	2	.
Smith..........Dade .................... Sun HilJ.......Wash’ton	200	3	1
Smith’s Mill  Jasper.................... SuDny Dale.. ..Chattooga 250 1  
Smithborough.. Jasper....	100......... Sunny Side.....Spalding.. 150	2 ...
Smithville.....Lee............. 3....... Surrency....... Appling.. 175	1 ...
Smyrna......... Cobb ...... 300	3	1 Suttalee......Cherokee. 150	1 ...
Snapfinger.....DeKalb................... Suwannee.......Gwinnett. 250	2 ...
Snap’ing Shoals Newton ...	200	3 ... Swainesborough Emanuel 300	2 ...
Snelson........Meriw’her 150............ Sycamore.......Irwin......	75.........
Sniff..........Coffee...	150......... Syllsfork......Ogleth’pe 200 ...........
Snow...........Dooly....	200	1	1	Sylvania......Scriven ...	500	3 ...
Social Circle..Walton...	800	2	.. Tails Creek....Gilmer...	75.........
Sonora.........Gordon...	50	1.... Talbotton......Talbot..... 1500	5	2
Sophia ........Bartow ...	100	1 ... Talking Rock.... Pickens................
Soque..........Hab’sham 300 ............ Tallapoosa.....Haralson.. 500 ..........
South Newport.. McIntosh 90............. Tallofcas......Brooks.....	240	2 ...
Sparta.........Hancock.	850	4	2 Tallulah......Rabun......	100........
Spoonville.....Houston .	300	1 ... Tarborough.....Camden ..	500	1 ...
Spread.........Jefferson.. 100.......... Tate...........Pickens... 150...........
Spring Place...Murray... 400	8 ... Tatum..........Forsyth... 75	1 ...
SpriDgfield....Effingh’m 100	2 ...[Taylor.........Crawford 350	1 ...
Table No. I.—Continued.
tn	cj	<n	cj
Sj S	cs5
® *O ® q	® "5 ® 'q
TOWN. COUNTY g ojj TOWN. COUNTY g s£ o 3
.2 SW	.2	©	tn<4
03	® •	=8	£	® _
■—	.	32 to	■—«	321©
£ 3	3	3
£_£	s	£	h	s
Taylor’s Creek.. Liberty ...	150	1.... Union..........Stewart...	100	1...
Taylorsville... Bartow ...	150	1.... Union Point....Greene.....	700	3 ..
Tazewelle...... Marion ...	200	3 ... Unionville.....Monroe...	100	I...
Teagle.........Gwinnett.	300 ....... Upatoie........Muscogee	150........
Teloga Springs.. Chattooga	300....... Upshaw.........Cobb.......	90........
Temperance..... Telfair...	100	1 ...... Urena.......Banks......	150........
Temple^........Carroll....	500	2 ... Valdosta....v.. Lowndes.. 2500	3 ..
Tennille....... Wash’ton	500	2	2	Valley Store.Chattooga	.	100	3 ..
Tesnatee....... White.....	100	1.... Vameville......Berrien ...	300 .......
Texas..........Heard......	150....... Van’s Valley...Floyd............ 2	....
Thad...........Chatt’ho’e	50	1.... Varnell’s Stati’n	Whitfield	100	1...
The Glades.....Hall.......	75	1.... Veazey.........Greene.....	125........
The Rock.......Upson ....... 200	1	.. Vernon.........Troup...................
Thomas Mills ... Floyd....	100	1.... Vickery’s Creek Forsyth... 125.........
Thomaston .....Upson...... 1000	4 ... Victory......... Carroll..	200	1	.
Thomasville....Thomas... 4000	13	7 Vienna........ Dooly............ 2....
Thomps’n’s Mis Jackson...	50....... Villa Rica.....Carroll....	300	1	1
Thomson ....... McDuffie..	800	6 .. Villanow ......Walker ...	80	4	.
Tickanetley.... Gilmer....	100........ Vineyard.......Spalding................
Tifton......... Berrien... 500 ........ Vining Station.. Cobb ..... 150	....
Tilly.......... Polk......	110........ Virgil.........Jackson... 75...........
Tilton......... Whitfield 250	1 --- Visage.........Towns......	200 .......
Timson......... Rabun.....	50........ vt acoville ...Haralson.. 200 .........
Toccoa......... Habers’m 1200	3 ... Wadley.........Jefferson. 300	3 ..
Toombsborough Wilki’son 150	1	1 Wahoo ........Lumpkin. 240 ...........
Toonigh........Cherokee..	200	6 ... Wainright......Chariton	150.......
Towaliga.......Butts......	125	2 ... Walden.........Bibb.......	100	1	1
Town Creek..... Gilmer,.... 100........ Walesca .......Cherokee.. 300 .........
Towns..........Telfair.... 115	1.... Walker Station Doug’erty..................
Trader’s Hill..Charlton..	150 ....... Walnut.........Jackson....	125	1...
Travis.........Habers’m	100....... Walnut	Hill.Franklin..	250 .... ...
Tray........... Habers’m ....... 1..... Walthourville... Liberty ...	400	2 ..
Trenton ....... Dade......	300	...... Wano...........Henry......	100........
Trible.........Clarke.....	75......... Ward Station... Randolph 300	3 ..
Trickum.......... Whitfield 100	2.... Waresborough.. Ware.......	100 ......
Trion Factory... Chattooga...	2.... Warm Springs.. Meriwet’r 250 ..........
Trip...........Gwinnett	100	1.... Warnerviile....Meriwet’r	200	1 ..
Troup Factory.. Troup.....	350	1	.. Warrenton .....Warren...	1100	1...
Tuckahoe.......Scriven ...	100	2 ... Warrior........Bibb.......	150	2 ..
Tugalo......... Habers’m	125	1.... Warsaw.........Milton.....	50 .......
Tunnel Hill.... Whitfield	300	2	.. Warthen........Wash’ton	300	3 ..
Turin.......... Coweta....	150	4	.. Warwick........ Worth........ 2........
Turkey Creek... Dooly.....	140........ Washington.....Wilkes..... 2400	7	3
Turnerville.... Habers’m	100	1.... Waterville.....Walker... 60............
Tusculum....... Effi’gham 200.......... Watkinsville...Oconee.....	250	4 ..
Tweed..........Laurens...	200 ....... Waverly Hall...	Harris..	500	1	.
Twiggsville.... Twiggs. ...	75	1.... Waycross.......Ware....... 2000	2	.
Twilight....... Miller....	150....... Wayn’sborough	Burke... 1600	5	1
Two Run........ Lumpkin ............... Wanesville.....Wayne......	250	2 ..
Ty-Ty.......... Worth........ 3........ Waynmanville.. Upson.-............. ...
Type...........Douglas... 90........... Way’s Station... Bryan........... J	......
Table No. I.—Continued.
crj c5	OS
S C5	§ O
ol 2o	0'S IE'S
TOWN. COUNTY g = ~ ° t TOWN. COUNTY g g£ ° I
.2	5	.2	0
-4J	.	4
4	-3	5
5	2s	ft	«
00“^	o	o
ft	ft	3	ft	ft	3
Webster Place.. Egbert...	100........ Wisdom’s Store. Harris....	250	1 ....
West Bowersv’le Franklin..	100	1.... Withers........Clinch.....	125.......
West Point.....Troup..... 2500	4	3 Wolf Creek....Wilcox....	200 .......
Weston......... Webster...	250	3 .... Wood Station... Catoosa ...	200	......
Westonia.......Coffee....	100........ Woodbury.......Meriw’h’r	200	2 ....
Wheaton........ Appling............... Woodlawn.......Murray... 100..........
Whigham........Decatur...	450	3	.. Woodstock......Cherokee.	100.......
White Plains...Greene....	400	2	.. Woodville......Greene.....	100	1.....
White Sul. Spgs Meriw’h’r 150	1.... Woolley’s Ford.. Hall.....	200	1 ....
Whitesborough. Carroll...	400	4	1 Wootten’s Mill.. Telfair.	50........
Whitesville....Harris....	150	3	.. Worthville.....Butts......	150.......
Whitfield...... Pulaski............... Wrightsville...Johnson...	450	7	2
WUiittington...Worth.....	100........ Wylly..........Laurens....... 2.......
Wier...........Lumpkin ............... Wynn’s Mill....Henry......... 1.......
Wildwood.......Dade......	100	1.... Yarborough.....Floyd......	75	1.....
Willacoochee.... Coffee............... Yatesville.....Upson......	100	2 ....
Williamsburg... Calhoun... 50......... Yellow Creek.... Dawson...............
Willingham Worth...................... Yellow Dirt Heard............ 1 
Wilsonville....Douglas...	160	1.... Yellow River...Gwinnett.	100	1.....
Winchester.....Macon.....	200	1 ... Yeomans........ Emanuel.	125	1.....
Windsor........Walton.... 90.......... York........... Houston.. 200	1 ....
Winfred........Jasper....	125........ Yorkville...... Paulding..	200 .......
Winn...........Douglas ............... Young Cane Union............. 1  
Winterville....Ogleth’pe.	225	1 ... Zebulon........Pike.......	250	3	1
Wiregaass......Clinch....	100........ Zoar...........Bulloch...	165.......
Table No. II.
This table shows the towns or districts grouped together ac-
cording to their population and the distribution of the physicians
among them. It also shows the number of members in each
group and the proportion that number bears to that of the phy-
sicians practicing in the group :
POPULATION NOT STATED.	POPULATION UNDER 400.
©s	©J
tn	PHYSICIANS. £ tj’i ra	PHYSICIANS. g
*	3	3	S*
pz	8	c	g
o	©	®	~ o	©	£
H	____ << P-___ H	g Uh
70	.............................. 46	No physician.	.......
37 1 Each .......... 37	  39	1	Each......... 39........
27 2 Each........... 54	  26	2	Each ........ 52........
8 3 Each .......... 24	  12	3	Each......... 36........
2	4 Each........... 8............ 7	4	Each ........ 28........
1	5 Each .......... 5	  4	5	Each......... 20........
--------------------------— ------ 1 6 Each ................ 6........
145	128	8	6 ----------------------------------
....—.....—	135	181	8	4
POPULATION UNDER 100.------------------------------------------
69 No physician.	....
36 1 Each .......... 36....... UNDER 390.
8 2 Each........... 16.......
2	3 Each .......... 6...........................................
2 4 Each............ 8.........   10	No physician.	.......
----------------------------------------6	1 Each........... 6........
115____________________66	5	7	7 2 Each .......... 14........
•-------------------------------------- 5	3 Each.......... 15........
UNDER 200	4	4 Each ........ 16........
________________________________________1	5 Each........... 5........
185 No physician.	  1	6 Each .......... 6........
133 1 Each.../...... 132............ 1	7 Each........... 7........
57 2 Each.......... 114	  1	8 Each .......... 8........
16 3 Each........... 48...........................................
5 4 Each .......... 20	  36	77	5	6
2 5 Each..... ..... 10....... —----------------------------------
2 6 Each ........... 6.......
399____________________331	9	2	UNDER 600.
UNDER 300.	----— —-----T— -------------------
__________________________________15 No physician.	.......
84 No physician.	. 11 1 Each... 11	.
71	1	Each.......... 71............ 9	2	Each ......... 18........
33	2	Each ......... 66	  9	3	Each.......... 27........
19	3	Each....4, ... 57	  4	4	Each ......... 16........
8	4	Each.......... 32	  4	5	Each......... 20........
2	5	Each.......... 10............ 1	7 ............... 7........
2	6	Each ......... 12............ 1	8 ............... 8........
219__________________248	9	3	54	  107	_7___6
Table No. II—Continued.
GO	OT
° ,g CD	2
cd	PHYSICIANS.	£	PHYSICIANS.	E
,Q ®	X> 0) E?
d wf3	O	d
O	|	H	|
H__________________________S Ph___________________________________S Ph
UNDER 700.	UNDER 40<;0.
2 No physician.	....... 1 6 ....................... 6.......
2	1 ...............  2........ 19 ........................ 9.......
1	2 ............... 2......... 1 10 ..................... 10.......
5	3 ............... 15	..........................................
1	4 ................ 4	..... 3	25	3	12
4 5 ................ 20	..... .............A	-------
15	43	6	14	UNDER 5000.
UNDER 800.	1 5 ...............~	5.......
____________________________________1 6 ....................... 6........
11 .................. 1	...... 19 ........................ 9.......
2	2 ................ 4	..... 1 12 ..................... 12.......
3	3 ................ 9........ 1 13 ..................... 13.......
2 6 ................ 12.............................................
------------------------------------ 5	45	18	40
8_____________________36	1___4	...... =
UNDER 900.	UNDER 6000.
1 No physician......................................................
1 2 ................. 2	..... 1 | 8...................... 8	|	4 | 50
1	3 ................ 3........ .....' ■	■
2	5 ..............  10........ UNDER 9000.
1 6 6 .................................................... ......- ..
------------------------------------1 | 9....................... 9	1	7 I 77
8	___________________29	8 27 ---------------------------------*—
UNDER 1000.	UNDER 10,000.
4	I 4 ....-r_-..... 16	I 2 I 12	1 I 12 .............. 12 I 7 53
UNDER 2000.
2 No physician................. OF 20,GOO INHABITANTS.
2 i ................. 2	...
2 2	.............. 4	1 | 30 .............. 30	| 4 13
9	3 ............... 27	..... ................
6	4 ............... 24	.....
8 5 ................ 40	..... 23,000 INHABITANTS.
1	7	..."	7	1 | 24 .............  24	| 19 | 79
-	30	100 — —34 003 INHABITANTS.
UNDER 3 ,00.________________________________________________
----1 9.	-----9--------	1 I 46 .............  46	I 20 | 43
2	3 ””""”""””7”"" 6
3	4 ............... 12	..... 41	000	INHABITANTS.
3	5 ................ 7	......
3	8 ............... 24	......
1	9 ................ 9	   50,000	INHABITANTS.
19____________________87	21	24	1 | 33 ............. 133	|~43 j 32
Table No. III.
NUMBER OF PHYSICIANS.
Population...............None.	12345678
Unknown.................... 70	37	27	8	2	1 .......... 145
Less than 100.............. 67	36	8	2	2 ............. 115
100 and less than 200..... 185	133	57	16	5	2	1 ...... 399
200	“	“	“	300........... 84	71	33	19	8	2	1 ...... 218
300	“	“	“	400 .......... 46	39	26	12	7	4	1 ...... 135
400	“	“	“	500........... 10	6	7	5	4	1	1	1	1	36
500	“	“	“	600........... 15	11	9	9	4	4	.. 1	1	54
600	“	“	“	700............ 2	2	2	5	1	4	  16
700 “ “ “ 800.......v................. 1 2 3 ... 2 ......... 8
800 “	“	“ 900............ 1	...	1	1	2	2	1 ........ 8
900 “	“	“ 1000.......................... 4	............ 4
1000 “	“	“ 2000........... 2	2296811 ................... 31
1169
____________________ 482	338 174 89	45	28__8___3_1 1169
1 able No. 3.—This table shows the number of physicians in
the several towns in the State according to their population up to
2,000. An examination of this table develops the extraordinary
fact that there are 482 districts without any physician. Two of
these have each a population of between 1,000 and 2,000.
RECAPITULATION OF TABLE 3.
1.	Population op Town or District.—Number of inhabitants
not given in the Gazetteer, 145; less than 100 inhabitants, 115;
less than 200 inhabitants, 399; less than 300 inhabitants, 218;
less than 400 inhabitants, 135; less than 500 inhabitants, 36; less
than 600 inhabitants, 54; less than 700 inhabitants, 16; less than
800 inhabitants, 8; less than 900 inhabitants, 8; less than 1,000
inhabitants, 4; less than 2,000 inhabitants, 31.
2.	Medical Service.—482 places have no physician, 338 have
one each, 174 have two each, 89 have three each, 45 have four
each, 28 have five each, eight have six each, three have seven
each, two have eight each.
Table	No. IV.
G	02	C
2	C	/	tl
*—	®S	u
TOWN OR CITY.	-S '5	© -	MEETINGS.
3	tn	-	©
5*	£	s
JL	h	2	1
Atlanta ...................... 50,000	133	43 32 1866,	1873, 1878 1882.
Savannah......................41,<'00	35	14 40 1869,	1875, 1885.
Augusta....................... 34.000	46	20 43 1868,	1876 1880.
Macon......................... 23 000	24	19 79 1870,	1877, 1884.
Columbus..................... 20,000	30	4 13 1872.
Rome.......................... 9 000	12	7 ox 1879.
Athens........................ 8,000	9	7 77 1883.
Americus..................... 5,000,	8	4 50 1871.
Albany........................ 4 000	5	3 60
Brunswick..................... 4 000	6	...
Griffin....................... 4 000	12	2 16 1867.
Milledgeville................. 4,000	9	6 66
Thomasville................... 4,000	13	7 53 1874, 1881.
Cartersville ................. 3.000	9	...
Dalton........................ 3,000	6	...
Marietta.-................... 3,<-00	10	1 10
Gainesville................... 2.750	5	1 20
LaGrange...................... 2,700	6	...
Madi‘on....................... 2,600	5	3 66
Cuthbert...................... 2,500	9	.
Hawkinsville.................. 2,500	8	5 62
Newnan........................ 2 5"0	8	2 25
Va dosta...................... 2 500	3	...
West Point.................... 2,500	4	3 75
Washington.................... 2,400	7	3 42
Barnesville................... 2 000	5...
Cedartown..................... 2,000	8	1 12
Covington..................... 2 000	3	1 33
Dawson........................ 2.000	4	..
Fort Valley................... 2.000	6	2 33
Quitman....................... 2,000	4	..
Waycross...................... 2,000	2	...
Tabic No. 4.—This table gives the names of towns of 2,000
inhabitants and upwards. The population, number of physi-
cians, number of members of the Association, percentage of
members to the number of physicians, and the dates of the meet-
ings of the Association.
An examination of this table shows that the Association has
held its meetings in ten places in which there is a total of 322 phy-
sicians, and that it draws from these 127 members equal to 39
per cent, of the physicians, while from the remaining 1,403 phy-
sicians in the State, the Association only gets 145 members, which
is very little over ten per cent. In other words, 46 per cent, of
the membership of the Association is drawn from the 322 physi-
cians practicing in the ten cities where the Association has met,
and 54 per cent, of the membership comes from 1,403 physicians
practicing in the rest of the State.
These figures fairly warrant the conclusion that the strength
of the Association will be principally derived from those places
in which its meetings are held, and the necessary conclusion fol-
lows that the more these are localized the more limited will be
the sphere of the Association and the smaller its membership.
It may be said that physicians practicing in isolated places are
more self-reliant than those residing in large communities, but
granting that they are so, they certainly are not unappreciative
of the advantages to be derived from intercourse with persons
pursuing the same line of thought.
These tables will be of value in selecting a place of meeting
for the Association. It will be seen that in Columbus, where the
Association has not met for fourteen years, there are 26 unaffili-
ated physicians out of a profession numbering 30. In Griffin,
where there has been no meeting since 1867, there are but t wo
members out of twelve physicians. Cartersville and Cuthbert
have nine physicians, each, and not a member in the Association,
and so on through the list.
In the above table I have given towns of 2,000 inhabitants,
because I think, that when the Association met at Griffin in 1867,
or at Thomasville in 1874, neither of these places could have
had much over 2,000 inhabitants, and I imagine that a town of
that size could comfortably accommodate the Association.
PROPOSED PLAN.
The feasibility of the following plan is dependent upon the cal-
culation that there will be remaining in the treasury a small sum
of money after paying all the expenses of the Association, as it
was said at the last meeting of the Association would be the re-
sult of adopting the present plan of publishing the proceedings;
1.	Let a committee, standing (for example, the Nominating
Committee) or special, report a place of meeting, in the selection
of which the committee will be guided solely by the interests of
the Association. Of course, such items as accessibility, accom-
modations, the number of physicians likely to be brought in con-
tact with the Association, etc., will be taken into consideration.
2.	Let the Secretary take charge of and be responsible for the
arrangements, and let him go or send to the place of meeting a
sufficient number of days before the time of assembling, secure
suitable rooms for the meeting of the Association, and make pro-
vision for the accommodation of the members.
3.	All expenses incurred by the Secretary in the discharge of
this duty to be paid by the Association.
4.	Let a circular be sent a month or so before the meeting to
every regular physician in the State, giving the time and place of
meeting, asking his co-operation, and setting forth the advantages
of being a member of the Association, and stating that even if
not a member, the Association would still be glad to receive from
him a paper, which could be presented through any member.
It is customary, I believe, to allow all regular practitioners to
take part in the scientific discussions, but not in the business af-
fairs of the body, therefore the presence of these gentlemen could
not fail to be agreeable and profitable as well to themselves as to
the members of the Association.
At present few physicians, outside its membership, know any-
thing of the time or place of meeting of the Association.
The Association has never taken any steps to bring itself to the
notice of isolated practitioners throughout the State. The Asso-
ciation cannot visit each individual, but it can send each one an in-
vitation to be present at the meeting. It can let itself be known
as a live, active body, seeking to advance medical unity and medi-
cal education, promote medical discussion and harmonize and con-
solidate the medical elements of the State, for mutual improve-
ment and mutual protection.
It is evident that the interests of the Association have suffered
from the practice of constantly meeting in the larger towns. By
meeting occasionally in the smaller places, it will become better
known to the hard working country practitioner, and the area of
its influence will be correspondingly extended.
The Association being no longer the guest of the physicians
living at the place of meeting, it follows that they could not feel
themselves obliged to provide entertainments, the expenses of
which bear heavily upon the few practitioners in smaller towns.
There is nothing in the plan, as set forth above, to prevent the
Association accepting the invitation of the faculty of any town
that desires to have the body as a guest.
These propositions are submitted purposely to provoke criti-
cism, in the hope that something better may be suggested, here
at least, we have certain data as a foundation for the discussion
of this matter at the next meeting. With these matters fairly
set before us now, we will at that time be better prepared to act
prudently and wisely upon this subject from which may come
so much of good or of evil to the Medical Association of Georgia.
Respectfully,	R. J. Nunn, M. D.
Savannah, Ga., Sept, i, 1885,
				

