# Correction

**Published:** 1885-12

**Authors:** 


CORRECTION.—In Dr. Nunn's letter in the October number of THE JOURNAL, he failed to credit Panola, DeKalb county, with one member of the Association.

